# Plate Wall Offset Measurement for U-Shaped Groove Workpieces Based on Multi-Line-Structured Light Vision Sensors

**DOI:** 10.3390/s25041018

**Published:** 2025-02-08

**Authors:** Yaoqiang Ren, Lu Wang, Qinghua Wu, Zhoutao Li, Zheming Zhang

**Affiliations:** 1 School of Mechanical Engineering, Hubei University of Technology, Nanli Road, Wuhan 430068, China; ryq7408@163.com (Y.R.); lpp3362@163.com (Z.L.); 13164124473@163.com (Z.Z.); 2 Hubei Provincial Key Laboratory of Modern Manufacturing Quality Engineering, Nanli Road, Wuhan 430068, China; 3 Hubei Institute of Aerospace Chemical Technology, Xiangyang 441053, China; wanglu@casc42.cn

**Keywords:** cylindrical shell assembly, plate wall offset measurement, multi-line structured light vision sensor calibration, local distance calculation between planes

## Abstract

To address the challenge of measuring the plate wall offset at the U-shaped groove positions after assembling large cylindrical shell arc segments, this paper proposes a measurement method based on multi-line-structured light vision sensors. The sensor is designed and calibrated to collect U-shaped groove workpiece images containing multiple laser stripes. The central points of the laser stripes are extracted and matched to their corresponding light plane equations to obtain local point cloud data of the measured positions. Subsequently, point cloud data from the plate wall regions on both sides of the groove are separated, and the plate wall offset is calculated using the local distance computation method between planes in space. The experimental results demonstrate that, when measuring a standard sphere with a diameter of 30 mm from multiple angles, the measurement uncertainty is ±0.015 mm within a 95% confidence interval. Within a measurement range of 155 mm × 220 mm × 80 mm, using articulated arm measurements as reference values, the plate wall offset measurement uncertainty of the multi-line-structured light vision sensor is ±0.013 mm within a 95% confidence interval, showing close agreement with reference values.

## 1. Introduction

Large cylindrical shells, with their high structural strength and stability, are commonly used as essential components in large equipment manufacturing. These shells consist of arcuate ribs and cylindrical shell rings. Before welding cylindrical shell rings, a plate wall offset inspection must be performed at the groove positions of the assembled arcuate shell segments [1]. The magnitude of the plate wall offset affects the assembly accuracy of the arc shell segments, and excessive differences can impact the shell’s pressure resistance capacity [2].

Currently, plate wall offset measurement at arc shell segment groove positions primarily relies on manual methods using tools such as feeler gauges, wedge gauges, and vernier calipers. While these methods can achieve certain measurement accuracy, their contact-based nature can lead to measurement errors and surface damage [3]. Manual measurement has low efficiency and accuracy, and introduces subjective factors that lead to inconsistent measurement standards. Contact-based measuring equipment such as coordinate measuring machines and portable articulated arms has limited working ranges and low efficiency, failing to meet on-site measurement requirements. Therefore, automated measurement with high precision, high efficiency, and consistent evaluation standards is essential.

Optical three-dimensional measurement technology has become a crucial dimensional measurement method due to its advantages of being non-contact-operated, non-destructive, highly efficient, highly automated, and capable of obtaining high-density point clouds [4]. The arc shell segment surfaces undergo special treatments, resulting in insufficient reflectivity, making passive optical measurements unable to reliably capture surface features [5]. Among active optical measurements, structured light projection measurement using area-structured light [6] is unsuitable due to limitations in light size, measurement time, and environmental requirements. Line-structured light vision sensors are less affected by environmental factors and offer fast measurement speeds, making them suitable for plate wall offset measurement. However, the measurement accuracy of single-line-structured light vision sensors is highly dependent on the measurement angle [7], obtaining accurate dimensions only when the light plane is completely perpendicular to the measured surface. Deng et al. [8] proposed the use of a robotic arm carrying a single-line-structured light vision sensor for scanning measurements. T. Stenberg et al. [9] used a robotic arm with a single-line-structured light vision sensor to scan and extract weld toe positions. Chen et al. [10] used a single-line-structured light vision sensor to scan welds and designed a deep learning network for weld seam segmentation, achieving narrow weld detection and positioning. While single-line-structured light scanning measurement avoids angle limitations and improves efficiency, it introduces new interference factors such as displacement errors that affect the measurement results. Currently, many scholars are researching multi-line-structured light vision sensors. Liu et al. [11] proposed a multi-line laser classification and light plane calibration method, while Sun et al. [12] used three-line-structured light vision sensor data as a reference, combined with single-line-structured light projected at arbitrary angles for 3D reconstruction. Zhang et al. [13] used multi-line-structured light vision sensors to measure train wheelset geometric dimensions.

This paper presents a multi-line-structured light vision sensor based on active optical measurement technology for cylindrical shell ring plate wall offset measurement. Compared to single-line-structured light vision sensors, multi-line-structured light vision sensors can simultaneously acquire profile information from multiple positions through capturing a single image. Additionally, the light planes are unified into the same coordinate system during system calibration [12,13,14,15], introducing no external errors, requiring no perpendicularity to the measured surface, and eliminating motion error influences, thereby achieving higher measurement accuracy and faster measurement speed.

## 2. Design of a Plate Wall Offset Measurement Method for U-Shaped Groove Workpieces

**Figure 1:**
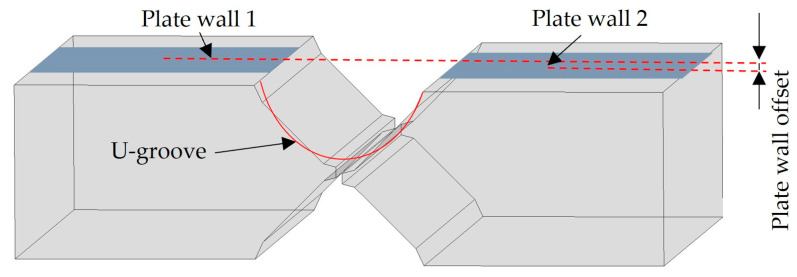
Schematic diagram of plate wall offset.

As illustrated in Figure 1, after the assembly of the arc shell segments, a height difference occurs between Plate wall1 and Plate wall2 on both sides of the U-shaped groove, which is referred to as the plate wall offset. The cylindrical shell rings require that the plate wall offset should not exceed 0.5 mm after assembly.

### 2.1. Measurement Principle of a Multi-Line-Structured Light Vision Sensor for Plate Wall Offset

**Figure 2:**
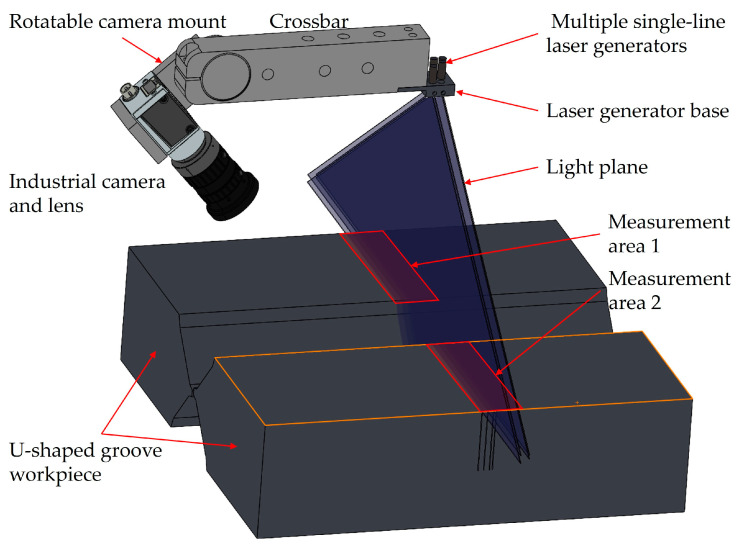
Schematic diagram of plate wall offset measurement using a multi-line-structured light vision sensor.

Plate wall offset measurement using a multi-line-structured light vision sensor is essentially based on the principle of triangulation [16]. As shown in Figure 2, multiple light planes are projected onto the measured area of a U-shaped groove workpiece, while a camera captures the laser stripe images modulated by the contours of the workpiece surface from another angle. The stripe images are processed to obtain point cloud data of the measured region’s surface for calculating the workpiece’s plate wall offset.

**Figure 3:**
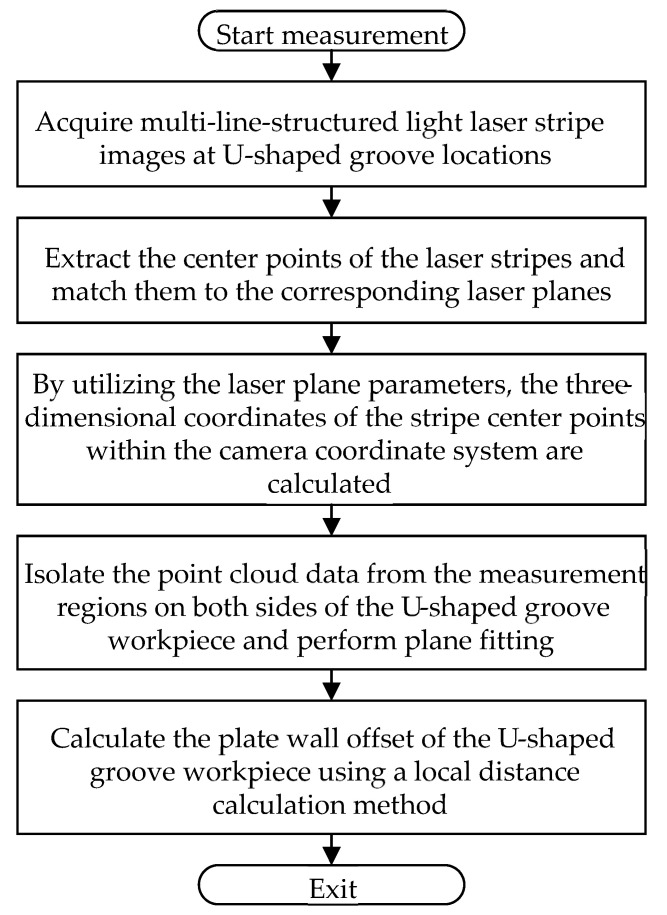
Flowchart of the plate wall offset measurement principle for U-shaped groove workpieces.

The principle of the plate wall offset measurement method is illustrated in Figure 3. The multi-line-structured light vision sensor captures laser stripe images from the U-shaped groove workpiece surface, and the laser stripe center points are extracted and classified to match their corresponding light plane equations. Then, using the mathematical model of the multi-line-structured light vision sensor, the three-dimensional coordinates of the point cloud data in the camera coordinate system are calculated for the measured area. Subsequently, the point cloud data for the plate wall regions on both sides are isolated. Plane fitting is performed separately on the point clouds of each plate wall region. Finally, the plate wall offset at the U-shaped groove position is calculated using the local distance calculation method between spatial planes.

### 2.2. Plate Wall Offset Calculation

**Figure 4:**
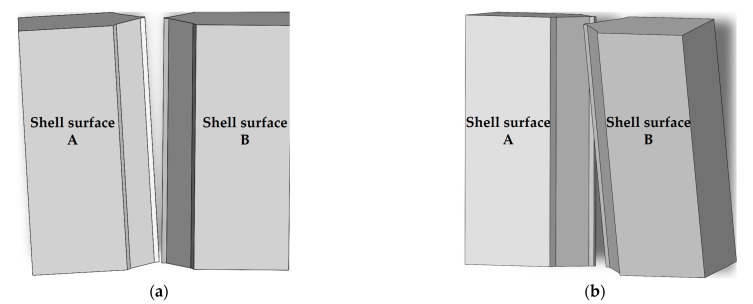
Schematic diagram of shell ring tilting. (**a**) Left–right tilting of the shell ring; (**b**) forward–backward tilting of the shell ring.

Due to the large volume of arc shell segments, the shell rings on both sides of the U-shaped groove can tilt forward–backward and left–right after assembly. As illustrated in Figure 4, Figure 4a depicts the left–right tilting at the point where shell surface A and shell surface B remain parallel. Figure 4b shows the forward–backward tilting where an angle exists between shell surface A and shell surface B.

Let Q represent the collected point cloud data of the workpiece surface, from which the point cloud data $Q_{1}$ and $Q_{2}$ are separated for the two measured regions. $Q_{1}$ contains m points and $Q_{2}$ contains n points.

Firstly, fitting is performed on $Q_{1}$ and $Q_{2}$. Due to the large diameter of the cylindrical shell rings, we can approximate the workpiece surfaces near the groove as planes for point cloud plane fitting. After fitting [17], two plane equations, $P_{1}$ and $P_{2}$, are obtained as shown in Equation (1).

(1) $$\left\{\begin{array}{c} P_{1}:a_{1}x+b_{1}y+c_{1}z+d_{1}=0\\ P_{2}:a_{2}x+b_{2}y+c_{2}z+d_{2}=0 \end{array}\right.$$

The parallel alignment of the shell surfaces on both sides of the U-shaped groove represents an ideal condition. However, in most cases, these two surfaces are not parallel. Given that two non-parallel planes in space must intersect along a line [18], the fitted plane equations cannot be directly used to calculate the plate wall offset. This paper adopts a local distance calculation method between spatial planes to calculate the plate wall offset.

The calculation process involves computing the distance from each point in $Q_{1}$ to plane $P_{2}$, and from each point in $Q_{2}$ to plane $P_{1}$, and then taking their average distance as the plate wall offset. The calculation process is as follows.

(a) Calculate the distance $d_{i}$ from each point $\left(x_{i},y_{i},z_{i}\right)$ in $Q_{1}$ to the fitted plane $P_{2}$, and obtain the average value $d_{1}$.

(2) $$d_{i}=\frac{\left|a_{2}x_{i}+b_{2}y_{i}+c_{2}z_{i}+d_{2}\right|}{\sqrt{a_{2}^{2}+b_{2}^{2}+c_{2}^{2}}}$$

(3) $$d_{1}=\frac{1}{m}\sum_{i=1}^{m}d_{i}$$

(b) Calculate the distance $d_{j}$ from each point $\left(x_{j},y_{j},z_{j}\right)$ in $Q_{2}$ to the fitted plane $P_{1}$, and obtain the average value $d_{2}$.

(4) $$d_{j}=\frac{\left|a_{1}x_{j}+b_{1}y_{j}+c_{1}z_{j}+d_{1}\right|}{\sqrt{a_{1}^{2}+b_{1}^{2}+c_{1}^{2}}}$$

(5) $$d_{2}=\frac{1}{n}\sum_{j=1}^{n}d_{j}$$

(c) Calculate the average distance d, which is the plate wall offset.

(6) $$d=\frac{d_{1}+d_{2}}{2}$$

The above analysis shows that the key to plate wall offset calculation lies in obtaining the workpiece surface point cloud data Q. The accuracy of point cloud data acquisition determines the measurement precision of the plate wall offset [19,20], making point cloud data acquisition using multi-line-structured light vision sensors one of the key technologies.

## 3. Measurement Model of a Multi-Line-Structured Light Vision Sensor

As shown in Figure 2, the multi-line-structured light vision sensor mainly consists of a crossbar, a rotatable camera mount, a laser generator base, an industrial camera, a lens, and multiple single-line laser generators.

**Figure 5:**
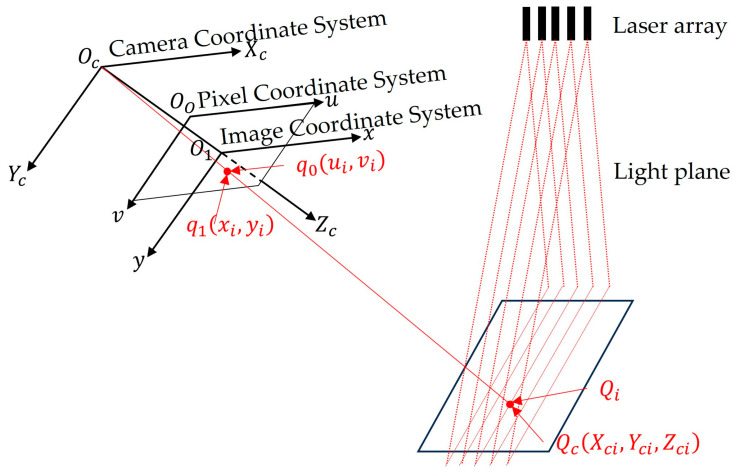
Measurement model of multi-line-structured light vision sensor.

For convenience of description, a sensor measurement model is established as illustrated in Figure 5 [21]. In this model, the camera coordinate system $O_{C}-X_{C}Y_{C}Z_{C}$ has its origin at the camera optical center $O_{C}$, with the $X_{C}$ axis along the horizontal direction of the image plane, $Y_{C}$ axis along the vertical direction, and $Z_{C}$ coinciding with the optical axis. The image coordinate system $O_{1}-xy$ has its origin at the intersection of the camera optical axis and image plane, forming a two-dimensional plane coordinate system parallel to the $X_{C}O_{C}Y_{C}$-plane. The pixel coordinate system $O_{0}-uv$ has its origin at the top-left corner of the image as the origin, which represents a two-dimensional coordinate system for point positions within the image.

Let the pixel coordinates of a laser stripe center [22] point $Q_{i}$ be $q_{0}(u_{i},v_{i})$, with the corresponding image coordinates $q_{1}\left(x_{i},y_{i}\right)$ and camera coordinates $Q_{c}\left(X_{ci},Y_{ci},Z_{ci}\right)$. The light plane equation is $A_{i}X_{c}+B_{i}Y_{c}+C_{i}Z_{c}+D_{i}=0$.

The calculation of the image coordinates $q_{1}\left(x_{i},y_{i}\right)$ from pixel coordinates $q_{0}(u_{i},v_{i})$ is

(7) $$x_{i}=u_{i}-u_{0}\;,\;y_{i}=v_{i}-v_{0}$$

The image coordinates $q_{1}\left(x_{i},y_{i}\right)$ are transformed to the normalized plane coordinates $q\left(x_{j},y_{j}\right)$.

(8) $$x_{j}=\frac{x_{i}}{f_{x}}\;,\;y_{j}=\frac{y_{i}}{f_{y}}$$

where the normalized plane is Z=1 in the camera coordinate system, which is a part of the camera coordinate system itself [23]. Here, $f_{x}$ and $f_{y}$ are the normalized focal lengths for the x-axis and y-axis, with $u_{0}$ and $v_{0}$ the pixel coordinates of the image center.

The light plane equation is used to calculate the corresponding camera coordinates $Q_{c}\left(X_{ci},Y_{ci},Z_{ci}\right)$ for the normalized plane coordinates $q\left(x_{j},y_{j}\right)$.

In the camera coordinate system, a point on the normalized plane extends into a ray along the $Z_{C}$ axis direction [24]. The camera coordinate $Q_{c}\left(X_{ci},Y_{ci},Z_{ci}\right)$ corresponding to the pixel coordinate point $q_{0}(u_{i},v_{i})$ is the intersection of this ray with the light plane.

(9) $$\left[\begin{array}{c} X_{ci}\\ Y_{ci}\\ Z_{ci} \end{array}\right]=-\frac{D_{i}}{A_{i}x_{j}+B_{i}y_{j}+C_{i}}\cdot \left[\begin{array}{c} x_{j}\\ y_{j}\\ 1 \end{array}\right]$$

Given $f_{x}$, $f_{y}$, $u_{0}$, $v_{0}$ and the light plane coefficients $A_{i}$, $B_{i}$, $C_{i}$, and $D_{i}$, the point cloud data of the object’s surface corresponding to the laser stripe center points can be obtained through Equations (7)–(9). The calibration of the sensor, which is equivalent to the mathematical model of multi-line-structured light vision sensor measurement, involves determining the parameters in Equations (7)–(9).

## 4. Sensor Calibration and Measurement Experiments

The experimental environment consisted of a Windows-based computer, an optical test platform, a single-axis height control platform, a single-axis high-precision displacement platform, and a 12 × 9 black-on-white ceramic chessboard calibration plate with 5 mm grid spacing and ±0.005 mm accuracy.

Based on the dimensions of the measured object, the groove top spacing was 80 mm with a depth of 40 mm. The selected camera model was a Basler acA3800-10gm (Basler GmbH, Aachen, Germany), with 3800 × 2748 resolution and 1/2.3″ chip size. The lens was an OPT 8 mm lens, which supports a maximum sensor size of 2/3″. According to the measurement requirements, with the laser generator base as the reference and the light plane perpendicular to the laser generator base, the optimal working distance was set to 170 mm, and the multi-line-structured light vision sensor was calibrated over a Z-axis range of 130–210 mm. Among them, the X-axis resolution was 0.033 mm/pixel, the Y-axis resolution was 0.033 mm/pixel, and the Z-axis resolution was 0.045 mm/pixel. The camera field of view was 120 mm × 108 mm at 130 mm and 155 mm × 220 mm at 210 mm, meeting the measurement field requirements.

### 4.1. Camera Calibration

Camera calibration is fundamental to measurement system calibration. The camera calibration accuracy affects both light plane calibration and system measurement accuracy. Re-projection error is the most commonly used method for evaluating camera calibration accuracy [11].

The camera was calibrated using Zhang’s calibration method [25], and a re-projection error of 0.13 pixels was achieved. The camera internal parameter K, radial distortion coefficients $K_{1},\;K_{2},\;K_{3}$, and tangential distortion coefficients $P_{1},\;P_{2}$ were obtained, with the calibration results shown in Table 1.

### 4.2. Light Plane Calibration

The multi-line-structured light vision sensor was horizontally fixed on a height control platform. After adjusting the height and the high-precision displacement platform position, the laser was activated to determine the calibration of the board’s position within the camera’s field of view, ensuring that all laser stripes fell within the calibration board image range for light plane calibration image acquisition.

Light plane calibration images are acquired in two steps [11]: first, capturing the calibration board image without laser projection, and then capturing the calibration board image with laser stripes, forming one set of images. Starting from 130 mm, images are captured at 10 mm intervals over nine steps until reaching 210 mm, collecting nine sets of light plane calibration images in total.

Light plane calibration mainly involves the conversion of light plane feature points from the pixel coordinate system, world coordinate system, and camera coordinate system [26].

World coordinates are calculated from pixel coordinates as follows:

(10) $$\left[\begin{array}{c} \begin{array}{c} X_{w0}\\ Y_{w0} \end{array}\\ Z_{w0} \end{array}\right]={\left(\left[\begin{array}{cc} K & 1 \end{array}\right]*\left[\begin{array}{cc} R & T\\ 0 & 1 \end{array}\right]\times \left[\begin{array}{c} \begin{array}{ccc} 1 & 0 & 0\\ 0 & 1 & 0\\ 0 & 0 & 0 \end{array}\\ \begin{array}{ccc} 0 & 0 & 1 \end{array} \end{array}\right]\right)}^{+}\cdot \left[\begin{array}{c} u\\ v\\ 1 \end{array}\right]$$

Camera coordinates are calculated from world coordinates as follows:

(11) $$\left[\begin{array}{c} \begin{array}{c} X_{c}\\ Y_{c}\\ Z_{c} \end{array}\\ 1 \end{array}\right]=\left[\begin{array}{cc} R & T\\ 0 & 1 \end{array}\right]*\frac{1}{Z_{w0}}\left[\begin{array}{c} \begin{array}{c} X_{w0}\\ Y_{w0}\\ 0 \end{array}\\ Z_{w0} \end{array}\right]$$

When the camera internal parameters, distortion coefficients, corner pixel coordinates $\left(u,v\right)$, and world coordinates $\left(X_{w},Y_{w},Z_{w}\right)$ are known, the rotation matrix R and translation matrix T can be solved via the PnP (Perspective-n-Point) algorithm [27].

**Figure 6:**
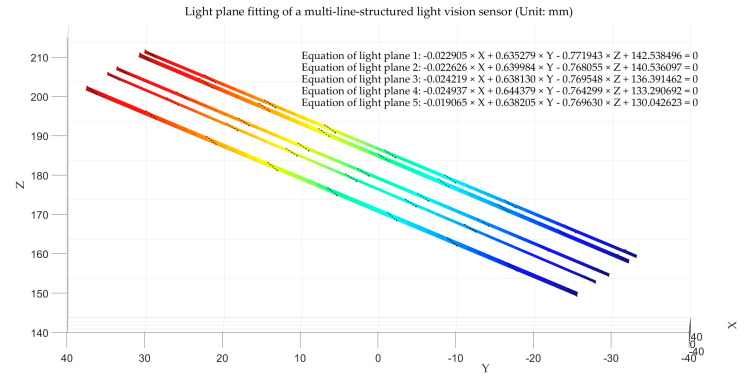
Light plane fitting of multi-line-structured light vision sensor.

Using Equations (10) and (11), the camera coordinate system’s coordinates of light plane feature points during calibration can be solved. Processing all nine sets of images sequentially yields the camera coordinates for all feature points. The light plane coefficients (A, B, C, D) in the camera coordinate system can be obtained by applying the least squares method to fit planes to the light plane feature points [11,13]. The calibrated light plane coefficients are illustrated in Table 2, and the fitted light planes are shown in Figure 6.

### 4.3. Sensor Calibration Accuracy Verification

#### 4.3.1. Standard Sphere Measurement Accuracy Verification

**Figure 7:**
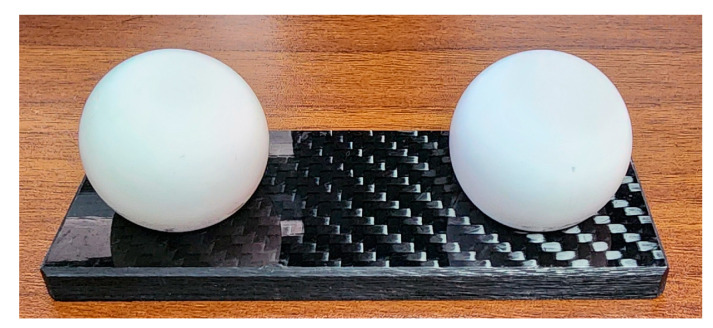
Standard sphere: 30 mm.

**Figure 8:**
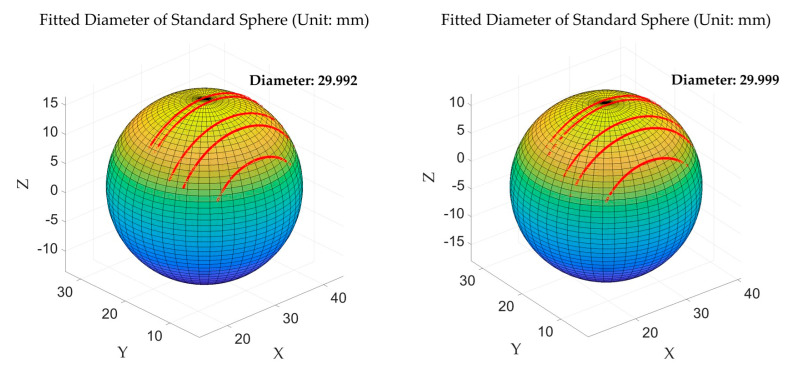
Standard sphere diameter fitting calculation.

To verify the calibration accuracy of the multi-line-structured light vision sensor, this study employed a standard sphere diameter fitting method. Measurements and diameter fitting calculations were performed on a high-precision ceramic standard sphere of known dimensions. The standard sphere is shown in Figure 7, while Figure 8 illustrates the diameter fitting schematic diagram for the standard sphere measurement using the multi-line-structured light vision sensor.

As shown in Figure 8, five contour points corresponding to light planes are clearly visible. Through sphere surface fitting based on the least squares regression [28] of these contour points, the sphere diameter was calculated and compared with the actual value. Each standard sphere was measured at five different angles, yielding a total of ten datasets. The actual diameter for all measurements was 30 mm, and the fitting results comparison is shown in Table 3.

**Figure 9:**
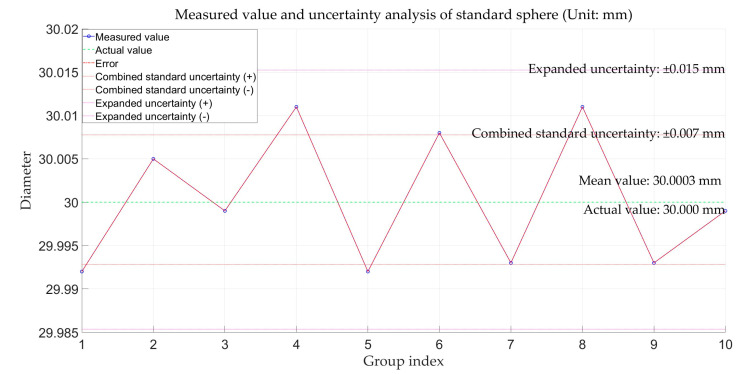
Measurement uncertainty analysis for standard sphere.

A measurement uncertainty analysis was conducted on the measurement results for the standard sphere diameter [29], with the results shown in Figure 9. It can be observed that the multi-line-structured light vision sensor calibrated in this study fell within the 95% confidence interval and achieved a measurement uncertainty of ±0.015 mm, which shows relatively high measurement accuracy.

#### 4.3.2. Flatness Measurement Experiment

The measurement object of this study is the height difference between the planes of the workpiece plate wall. As the flatness of point cloud data affects the calculation accuracy of plate wall offset, it is essential to ensure high flatness in the point cloud data collected by the multi-line-structured light vision sensor. Therefore, point cloud flatness measurement experiments need to be conducted first for the multi-line-structured light vision sensor.

**Figure 10:**
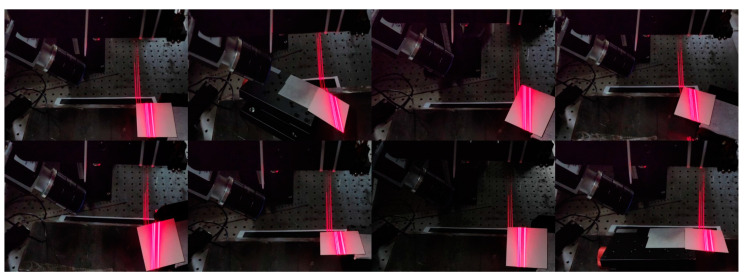
Multi-angle point cloud flatness assessment.

**Figure 11:**
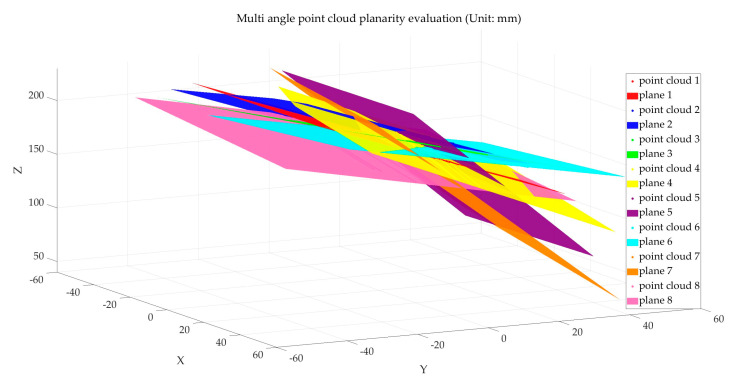
Multi-angle point cloud flatness assessment fitting schematic.

Using a ceramic calibration board as the measurement object, the plate was positioned appropriately at different angles relative to the light planes. The multi-line-structured light vision sensor was applied to capture images of the calibration plate in eight different poses, as shown in Figure 10. Polyworks software 2023 was used to evaluate the point cloud flatness. The flatness evaluation results are shown in Table 4, and the fitting schematic is illustrated in Figure 11.

The analysis of the error data in Table 4 shows an average error of 0.032 mm, with an uncertainty ±0.002 mm within a 95% confidence interval, indicating that the point cloud obtained by the multi-line-structured light vision sensor achieves high flatness.

#### 4.3.3. Comparison and Verification of Plate Wall Offset Measurement Results

**Figure 12:**
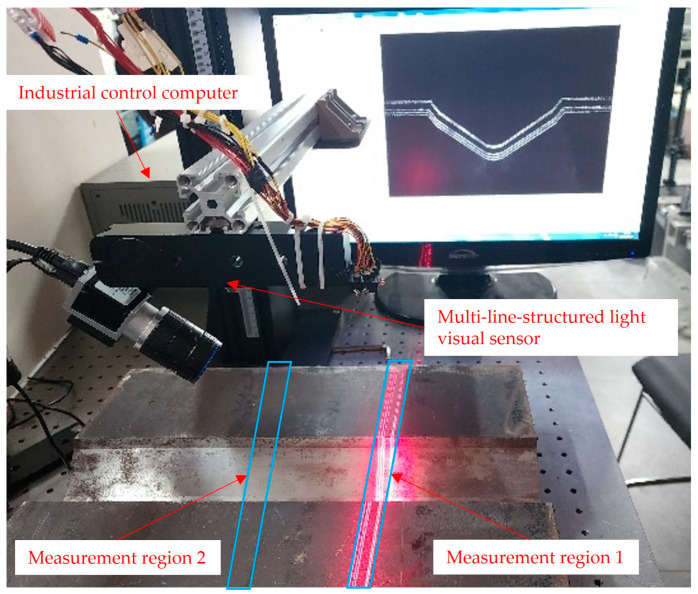
Multi-line-structured light visual sensor measurement experimental setup.

To verify the measurement accuracy of the multi-line-structured light vision sensor calibrated in this paper for plate wall offset detection, the detection results of the FARO flexible articulated arm (model FARO-Quantum-M) were used as the reference standard. The plate wall offset measurement setup was constructed as shown in Figure 12.

**Figure 13:**
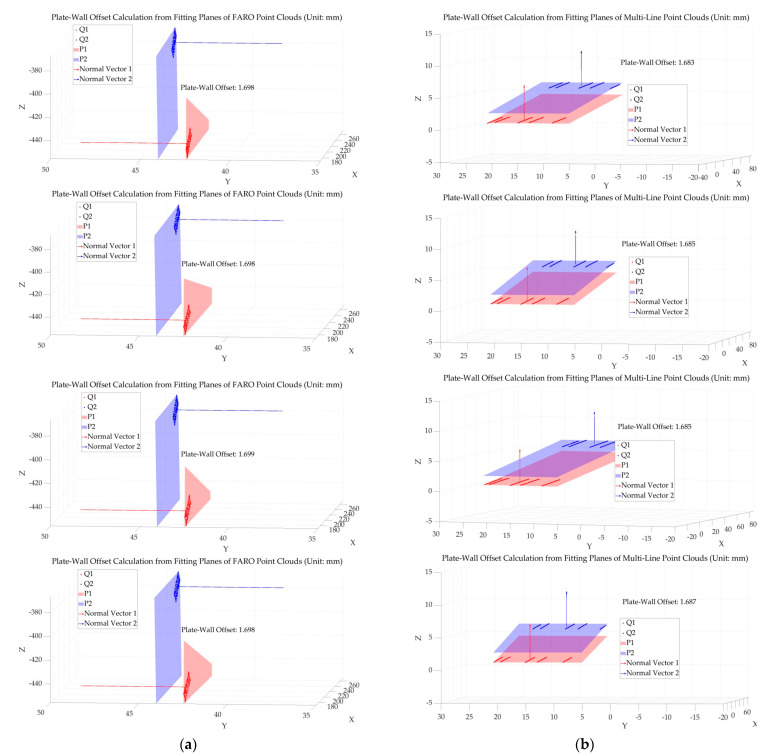
Comparison of plate wall offset measurement results. (**a**) Plate wall offset measurement results using the FARO flexible articulated arm; (**b**) plate wall offset measurement results using the multi-line-structured light sensor.

The U-shaped groove workpiece was placed on the optical experiment platform. Data acquisition of the region under investigation was performed using both a FARO flexible articulated arm and a multi-line-structured light vision sensor. For measurement region 1 and measurement region 2, as shown in Figure 12, the multi-line-structured light vision sensor and the flexible articulated arm were used to collect local point cloud data from five different angles for each region. A total of ten datasets were obtained from the two measurement regions. The acquired point cloud data were processed to calculate the plate wall offset. The results are illustrated in Figure 13, where Figure 13a represents the plate wall offset measured by the FARO arm, and Figure 13b shows the results obtained by the multi-line-structured light sensor. The measurement results of the ten datasets are presented in Table 5. From the error values in the table, it can be observed that the measurement results from the five angles at each measurement position are generally consistent, showing good measurement stability. The comparison error values across the ten datasets were within 0.020 mm. The average error value was −0.011 mm, and the uncertainty within a 95% confidence interval was ±0.013 mm, which satisfies the required measurement accuracy. Additionally, the single measurement time for each measurement was less than 200 ms, indicating a high measurement speed.

## 5. Conclusions

This paper addresses the challenges of high difficulty and insufficient accuracy in measuring the plate wall offset of large cylindrical shell plates. A method based on a multi-line-structured light vision sensor is proposed, providing an effective solution for measuring the U-shaped groove plate wall offset.

The multi-line-structured light vision sensor achieved the precise measurement of the stripe-covered area by unifying multiple light plane data within the same coordinate system. A multi-line-structured light vision sensor was used to calculate the plate wall offset measurement, eliminating the angular requirements of fixed single-line-structured light methods and the interference of displacement errors in scanning measurement. It offers faster measurement speed and higher measurement accuracy. This sensor can also be applied to the objects’ dynamic three-dimensional measurements, successfully collecting 3D object dimensions without interfering with object motion.

The plate wall offset measurement method designed in this paper, based on the multi-line-structured light vision sensor, obtains the measurement by collecting the local point cloud data of the measured object. This measurement method performs well on objects with regular surface morphology and good surface accuracy. However, for objects with complex surface morphology or poor surface accuracy, the measurement precision may be significantly affected. Future improvements could involve increasing the number of cameras to enhance the sensor’s viewing angle and adding more light planes to collect additional point cloud data. The measurement accuracy is expected to improve with the increase in point cloud data.

## Figures and Tables

**Table 1:** Camera parameters.

Parameter Name	Calibration Results
Camera Internal Parameters	$K=\left[\begin{array}{ccc} 5210.87655 & 0 & 1870.68786\\ 0 & 5210.43327 & 1302.10518\\ 0 & 0 & 1 \end{array}\right]$
Radial Distortion	$K_{1}=-0.06574,\;K_{2}=0.08895,\;K_{3}=0.00000$
Tangential Distortion	$P_{1}=0.00038,\;P_{2}=-0.00061$

**Table 2:** Light plane coefficients of the multi-line-structured light visual sensor.

Light Plane Index	1	2	3	4	5
Parameter A	−0.022905	−0.022626	−0.024219	−0.024937	−0.019065
Parameter B	0.635279	0.639984	0.638130	0.644379	0.638205
Parameter C	−0.771943	−0.768055	−0.769548	−0.764299	−0.769630
Parameter D	142.538496	140.536097	136.391462	133.290692	130.042623

**Table 3:** Comparison of fitted diameter results for the standard sphere (unit: mm).

Ceramic Standard Sphere Measurement	Angle 1	Angle 2	Angle 3	Angle 4	Angle 5
Standard value of Sphere 1	30.000	30.000	30.000	30.000	30.000
Measured value of Sphere 1	29.992	30.005	29.999	30.011	29.992
Standard value of Sphere 2	30.000	30.000	30.000	30.000	30.000
Measured value of Sphere 2	30.008	29.993	30.011	29.993	29.999

**Table 4:** Planarity evaluation results (unit: mm).

Serial Number	1	2	3	4	5	6	7	8
Planarity Error	0.031	0.033	0.032	0.032	0.033	0.031	0.032	0.033

**Table 5:** Comparison of plate wall offset measurement results (unit: mm).

Group Number	FARO Measured Value	Multi-Line Measured Value	Error Value
1	1.698	1.683	−0.015
2	1.704	1.695	−0.009
3	1.699	1.685	−0.014
4	1.698	1.687	−0.011
5	1.698	1.685	−0.013
6	1.559	1.566	0.007
7	1.558	1.543	−0.015
8	1.560	1.546	−0.014
9	1.559	1.542	−0.017
10	1.560	1.550	−0.010

## Data Availability

Data are contained within the article.
